# An LC Wireless Microfluidic Sensor Based on Low Temperature Co-Fired Ceramic (LTCC) Technology

**DOI:** 10.3390/s19051189

**Published:** 2019-03-08

**Authors:** Yongyuan Liang, Mingsheng Ma, Faqiang Zhang, Feng Liu, Zhifu Liu, Dong Wang, Yongxiang Li

**Affiliations:** 1 CAS Key Laboratory of Inorganic Functional Materials and Devices, Shanghai Institute of Ceramics, Chinese Academy of Sciences, Shanghai 200050, China; liangyongyuan@student.sic.ac.cn (Y.L.); zhangfq@mail.sic.ac.cn (F.Z.); liuf@mail.sic.ac.cn (F.L.); wangd@mail.sic.ac.cn (D.W.); 2 Center of Materials Science and Optoelectronics Engineering, University of Chinese Academy of Sciences, Beijing 100049, China; 3 School of Engineering, RMIT University, Melbourne VIC 3001, Australia; yongxiang.li@rmit.edu.au

**Keywords:** microfluidics, LTCC, wireless sensor, liquid sensing

## Abstract

This work reports a novel wireless microfluidic biosensor based on low temperature co-fired ceramic (LTCC) technology. The wireless biosensor consists of a planar spiral inductor and parallel plate capacitor (LC) resonant antenna, which integrates with microchannel bends in the LTCC substrate. The wireless response of the biosensor was associated to the changes of its resonant frequency due to the alteration in the permittivity of the liquid flow in the microchannel. The wireless sensing performance to different organic liquids with permittivity from 3 to 78.5 was presented. The measured results are in good agreement with the theoretical calculation. The wireless detection for the concentration of glucose in water solution was investigated, and an excellent linear response and repeatability were obtained. This kind of LC wireless microfluidic sensor is very promising in establishing wireless lab-on-a-chip for biomedical and chemical applications.

## 1. Introduction

Microfluidics is the science and technology that manipulates small amounts of liquid using channels with small dimensions [1]. Microfluidic systems offer advantages such as small sample volumes, low energy consumption, fast response times, and easy disposability, which enable microfluidic systems to serve as an important tool in the frontiers of medical science, biological analysis, pharmacy, chemistry, and environment [2]. The main application that drives the development of microfluidics is the point-of-care-test (POCT), which is defined as diagnostic testing at or near the site of patient care [3,4,5,6,7,8]. Various liquid sensing is one of the vital challenges for the POCT microfluidic system [9]. However, most of the reported microfluidic sensors utilize electrochemical or optical methods to monitor the changes of the liquids, which are usually accompanied by uncontrollable chemical reactions, fluorescent labelling, and expensive professional equipment.

Microfluidic sensors based on radio frequency (RF) or microwave detection techniques have attracted extensive attention in recent years since they are label-free, non-invasive, portable, and cost-effective [10,11,12,13,14,15]. It is noticeable that all of them need to connect with coaxial transmission lines or waveguides to acquire the testing signal, which makes them complex and cumbersome. With rapid development of wireless communication technology, and the emerging need for mobile healthcare, combining wireless technology with microfluidic sensors is of great significance [16]. The wireless sensors based on inductive-capacitive (LC) resonant antenna have been successfully used for measuring biological parameters. Rogers et al. introduced an ultrathin, stretchable LC based wireless microfluidic sensor for the measurement of the volume and chemical properties of sweat [17,18]. Boutry et al. presented a flexible LC sensor for wireless monitoring of blood flow in their latest report [19]. These works indicated that wireless sensors based on LC resonant antenna could be a promising method for developing wireless microfluidic sensors. On the other hand, most of the current microfluidic sensors use polymer substrates that are low cost in manufacturing, and suitable for flexible and wearable electronics. However, polymer materials suffer from poor chemical stability due to some organic and corrosion liquids. In addition, it is difficult for polymer materials to realize embedded electronic circuits with microchannel bends. Therefore, searching for substrate materials with good chemical inertness, that allow for low cost and easy integration with electronic circuits in the microfluidic devices, have been of continuing interest. 

Low temperature co-fired ceramic (LTCC) technology enables the fabrication of three-dimensional ceramic structures with embedded circuits, microchannel bends, and cavities. In particular, the good mechanical and electrical properties of LTCCs as well as its compatibility with thick film hybrid technology, makes it exceptionally suited for integrated sensors, and RF application [20]. LTCC technology can potentially satisfy the requirements of LC wireless microfluidic sensors including miniature size, ease of constructing microchannel bends, and compatibility with printed circuit technologies. A variety of LTCC-based microfluidic devices have been reported in applications, such as biosensors, bioreactors, cell monitors, and point-of-care diagnostic systems [21,22,23,24]. However, the development of LC wireless microfluidic sensors based on LTCC are still rare. 

In this work, a kind of LC wireless microfluidic sensor based on LTCC technology was developed. The wireless response towards different organic liquids and different concentrations of glucose in water solution was investigated. The possible sensing mechanisms of the developed sensors were discussed. This work provides a fast, convenient, and wireless sensor for continuous detection of liquids.

## 2. Sensor Design

**Figure 1:**
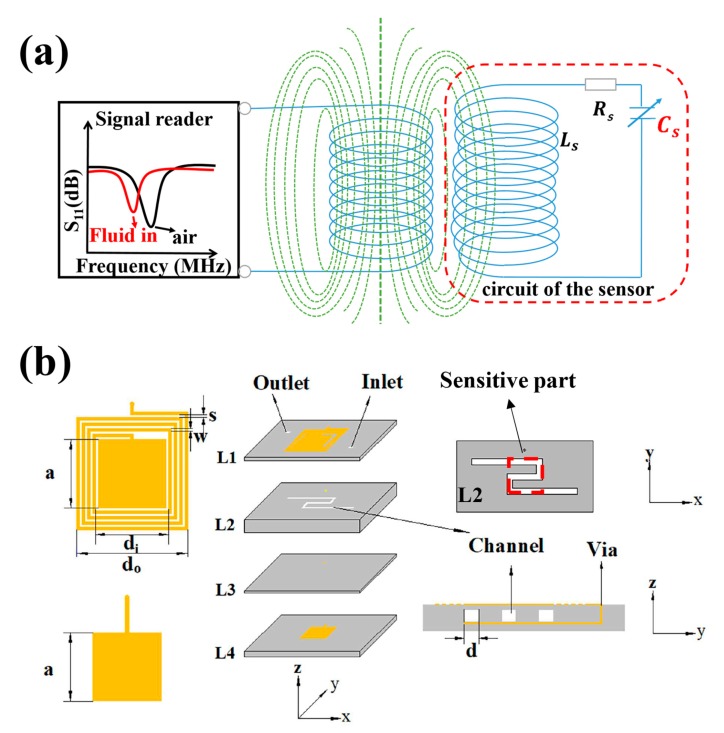
(**a**) Equivalent circuit of inductive coupling between the wireless microfluidic sensor and the reader antenna. (**b**) Schematic diagram of the designed sensor.

The wireless signal transmissions between the LC wireless microfluidic sensor and the external reader antenna can be described using a mutual inductance coupling circuit model, as shown in Figure 1a. The equivalent input impedance ($Z_{\mathrm{in}}$) of the reader antenna and the return loss (S11) of the coupling signal can be expressed as follows [25]:

(1) $$Z_{\mathrm{in}}=R_{0}+j2\pi L_{0}\left[1+\frac{k^{2}{\left(\frac{f}{f_{r}}\right)}^{2}}{1+\frac{j}{Q}\left(\frac{f}{f_{r}}\right)-{\left(\frac{f}{f_{r}}\right)}^{2}}\right]$$

(2) $$S_{11}=\frac{Z_{\mathrm{in}}-Z_{0}}{Z_{\mathrm{in}}+Z_{0}}{|}_{Z_{0}=50\Omega }$$

where Q is the quality factor of the LC circuit, k is the coupling coefficient between the LC microfluidic sensor and the external reader antenna. $Z_{0}$ is the intrinsic impedance of the signal emitter, with a fixed value of 50 Ω. Equations (1) and (2) indicate that the external reader antenna can form strong inductive coupling with the LC sensor when $f=f_{r}$, and the magnitude of $S_{11}$ drops to the minimum at the resonant frequency $f_{r}$, making sensing signals wirelessly detected.

The designed LTCC wireless microfluidic sensor is shown in Figure 1b. A planar square spiral inductor (*L_S_*) and a parallel plate capacitor (*C_S_*) with microchannel bends in the middle were integrated using the LTCC multilayer ceramic process. The inductor and capacitor connect through via the LTCC substrate to form a resonant circuit. The resonant frequency $f_{r}$ of the LC circuit is related to $L_{s}$ and $C_{s}$ values, as shown in Equation (3). *L_S_* and $C_{s}$ depend on the structural dimensions of the LC circuit with respective to Equations (4) and (5) [26].

(3) $$f_{r}=\frac{1}{2\pi \sqrt{L_{S}C_{S}}}$$

(4) $$L_{S}=1.39\times 10^{-6}\left(d_{o}-d_{i}\right)N^{5/3}\log\left(4\frac{d_{o}+d_{i}}{d_{o}-d_{i}}\right)$$

(5) $$C_{S}=\frac{\varepsilon_{0}\varepsilon_{eff}S}{D}$$

where $d_{o}$ and $d_{i}$ are the external and internal dimensions of the planar spiral inductor, respectively, N is the number of turns, $\varepsilon_{0}$ is free space permittivity (8.854 $\times 10^{-12}\;F/m)$, S is the area of the plate capacitor, *D* represents the distance between the two capacitor electrodes, $\varepsilon_{eff}$ is the effective permittivity of the dielectrics between the two parallel capacitive plates, including the parts of L1, L2, and L3. Consequently, the changes of fluids in the microchannel lead to change of $f_{r}$, which can be wirelessly detected by measuring the impedance variation of the reader antenna. For the composite between the two capacitor electrodes, as shown in Figure 1b, the parts of L1 and L3 are LTCC substrates, the part of L2 is a LTCC substrate with microchannel bends. L1, L2, and L3 are considered as a series model, and $\varepsilon_{eff}$ can be expressed as [27]:

(6) $$\frac{1}{\varepsilon_{eff}}=\sum_{1}^{k}\frac{v_{i}}{\varepsilon_{i}}=\frac{t_{1}+t_{3}}{\left(t_{1}+t_{2}+t_{3}\right)\varepsilon_{1}}+\frac{t_{2}}{\left(t_{1}+t_{2}+t_{3}\right)\varepsilon_{m}}$$

where $t_{1}$, $t_{2}$, $t_{3}$ are the thicknesses of L1, L2, and L3, respectively. $\varepsilon_{1}$ is the permittivity of the LTCC substrate (6.2), $\varepsilon_{m}$ is the effective permittivity of the sensitive part in L2, as shown in Figure 1b. The parameters of the designed LC microfluidic sensor are summarized in Table 1.

The effective permittivity of the two-phase mixture can be calculated by the semi-empirical formula [28]. The value of $\varepsilon_{m}$ is obtained by Maxwell–Garnett equation:

(7) $$\frac{\varepsilon_{m}-\varepsilon_{1}}{\varepsilon_{m}+2\varepsilon_{1}}=\nu_{2}\frac{\varepsilon_{2}-\varepsilon_{1}}{\varepsilon_{2}+2\varepsilon_{1}}$$

where $\varepsilon_{1}$ and $\varepsilon_{2}$ are the permittivity of the LTCC substrate and fluid filled in the microchannel, respectively. $v_{2}$ is the volume fraction of the fluid phase in two-phase mixed media, which was ~50% for the developed sensor. According to Equation (4), the calculated value of *L_S_* is 0.47 μH. The calculation of the *C_S_* value is related to the media filled in the microchannel. When the microchannel is filled with air ($\varepsilon_{2}=1$), the calculated *C_S_* value is 2.61 nF, according to Equations (5) and (6), and the calculated resonant frequency of the sensor is 143.48 MHz.

## 3. Sensor Fabrication and Test

**Figure 2:**
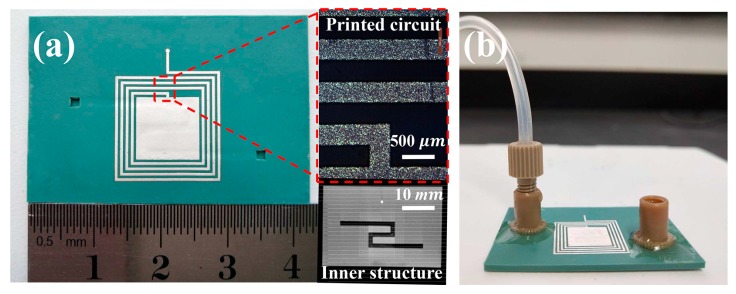
(**a**) Photographs of the sensor fabricated through the low temperature co-fired ceramic (LTCC) process. (**b**) The sensor mounted with connectors.

The sensors were fabricated using the LTCC process. The LTCC materials (SICCAS-K5F3) which had a dielectric constant of $\varepsilon_{r}$ = 6.2 and a dielectric loss tangent of tanδ = 0.001 (@10 GHz) were produced in our laboratory, and the details can be found in our previous paper [29]. Silver paste (Dupont, LL612, Wilmington, DE, USA) was used for preparing LC antenna for screen-printing (KEKO, P-200A, Zuzemberk, Slovenia). The fabrication of microchannel bends was a particularly important process for the LTCC microfluidic sensor. Most of the reported works used sacrificial volume materials (SVM) to achieve uniform microchannel structure in the LTCC body [22,24]. Here, we used an SVM-free process to construct the microchannel [30]. Firstly, as shown in Figure 1b, the LTCC green tapes with 5, 20, 1, and 10 layers, were stacked to form L1, L2, L3 and L4, respectively. The laminating pressure used was 5 MPa, and the laminating temperature was 60 °C with a holding time of 120 s. Secondly, L1 and L2 were laminated together to form L12 by using the same laminating conditions mentioned above. L3 and L4 were also laminated in this way to form L34. Finally, L12 and L34 were laminated together with the optimized laminating pressure, temperature, and time (5 MPa, 55 °C, 60 s), to form the microchannel structure in the LTCC body. The fabricated green samples were initially debinded at 450 °C for 120 min to remove the organic additives, and then they were sintered at 900 °C for 30 min in air. Liquid flow connectors were glued onto the surface of the sensor to ensure the fluids could pass through the microchannel smoothly. The internal microchannel bends were observed by X-ray non-destructive inspection (YXON, Y. CT Solution, Hamburg, Germany). Figure 2 shows the photographs of the sensor sample fabricated through LTCC technology. The size of the sensor was approximately 40 mm × 25 mm × 1.6 mm. X-ray images indicated that the obtained microchannel showed no obvious signs of sagging and delamination. The width and height of the microchannel were about 1.6 mm and 0.8 mm, respectively.

**Figure 3:**
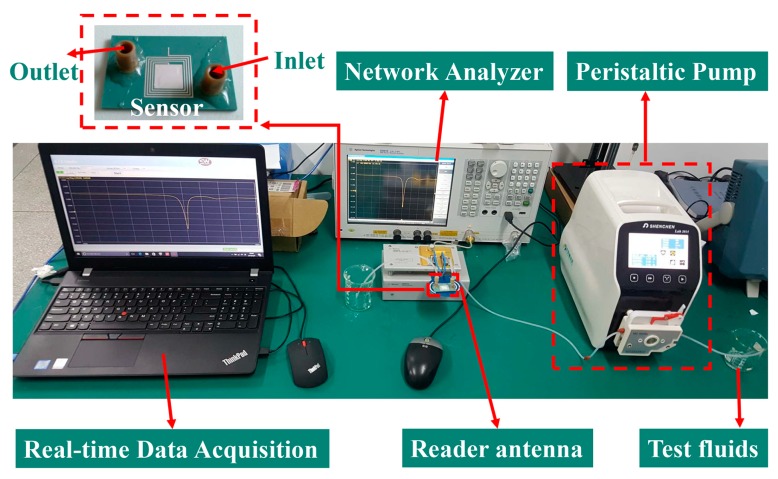
Testing platform for the inductive-capacitive (LC) wireless microfluidic sensor.

The testing platform for the wireless microfluidic sensor is shown in Figure 3. The reader antenna was connected to a vector network analyzer (Agilent, E5061B, Santa Clara, CA, USA). In order to extract the wireless signal steadily during each measurement, the LC sensor was directly placed onto the reader antenna. It should be mentioned that based on the near field coupling principle, the distance between the LC sensor and the external reader antenna was kept short (within 1 cm). The wireless signals would have degraded with an increase of distance. A peristaltic pump (Shenchen, Lab 2015, Baoding, China) was used for making the fluids flow into the microchannel evenly. Several typical liquids, cyclohexane, dichloromethane, aether, acetic acid, isopropanol, ethanol, aqueous ethanol solution, and deionized water were tested. The target liquids were pumped into the sensor and held steadily for 1 min before data were collected. The wireless signal was collected using real-time data acquisition software. After the completion of each measurement, ethanol was used to flush the microchannel followed by drying at 50 °C for 20 min to ensure the resonant frequency value of the sensor recovered to the original value. The dielectric properties of the tested fluids were measured by a coaxial probe measurement kit (Agilent, N1500A, Santa Clara, CA, USA). Since the coaxial probe method could cause obvious uncertainty in the measurement of dielectric properties, when the tested frequency was below 500 MHz [31], the tested frequency for the measurement of liquid permittivity in this work was 500 MHz.

## 4. Results and Discussion

**Figure 4:**
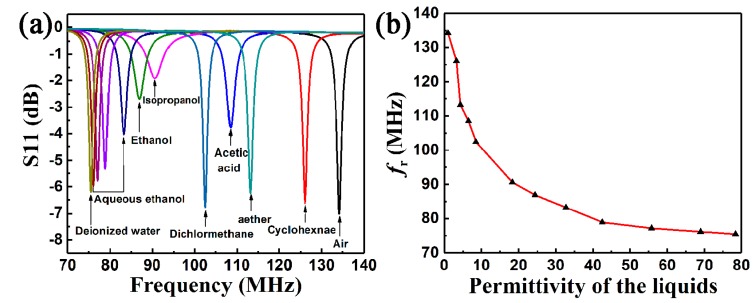
(**a**) Wireless signal response of the sensor to different kinds of organic liquid. (**b**) The relationship between measured $f_{r}$ and corresponding permittivity of the tested organic liquids.

Figure 4a shows the wireless signal response of the sensor to different kinds of organic liquid. When the microchannel was filled with air, the measured $f_{r}$ of the LC wireless microfluidic sensor was 134.20 MHz. The sensor presented significantly different frequency responses to these liquids, which was caused by the change of liquid permittivity. Figure 4b shows the function of the resonant frequency $f_{r}$ to measured permittivity of the tested liquids; $f_{r}$ decreased with increases in permittivity. This can be attributed to the increase of equivalent capacitance in the microchannel area according to Equations (3)–(6). It is worth noticing that the frequency response of the sensor showed a high sensitivity to different liquids. By introducing deionized water into the microchannel, $f_{r}$ decreased from 134.20 to 75.45 MHz, and $\Delta f_{r}/f_{r}$ was approximately 44%, which is much higher than that of the previously reported work using microwave ring resonators [32,33], T-resonators, and T-resonators integrated with radio frequency identification (RFID) chips to achieve wireless fluid sensing [34], and substrate integrated resonators [35,36,37]. Moreover, the sensitivity was much higher in low permittivity regions than in high permittivity regions since the slope of the curve in Figure 4b represents sensitivity of $f_{r}$ to the permittivity of fluids. The amplitude of $S_{11}$ had fluctuation with different fluids in the fluidic channel, which might have been due to the different dielectric losses of different liquids [32,34]. The non-polar solvents like cyclohexane had very low dielectric losses at the testing frequency range, and thus showed strong responses.

**Figure 5:**
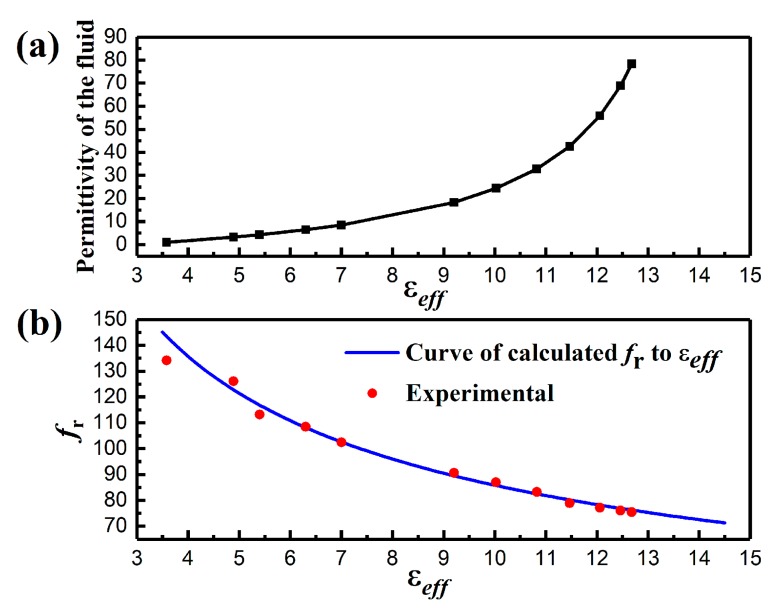
(**a**) Relationship between the calculated effective permittivity and the permittivity of the liquid. (**b**) Calculation of the resonant frequency to the effective permittivity, compared with experimental.

The function between $\varepsilon_{2}$ and $\varepsilon_{eff}$ is shown in Figure 5a. The variation of $\varepsilon_{eff}$ is much more significant for low $\varepsilon_{2}$ regions than that of high $\varepsilon_{2}$ regions. This implies that the wireless microfluidic sensors would show relatively high sensitivity when measuring low permittivity liquids. The relationship between the measured $f_{r}$ as a function to the calculated $\varepsilon_{eff}$ was also obtained, which presented good agreement with the curve of calculated $f_{r}$ versus $\varepsilon_{eff}$, as shown in Figure 5b. It indicates that the sensor had sensitive responses to the tested liquids in a wide range of permittivity. When the microchannel was filled with air, the measured resonant frequency was 134.2 MHz, which is smaller than the calculated value of 143.48 MHz. However, when the microchannel was filled with acetic acid ($\varepsilon_{2}=6.47)$, which was close to that of LTCC substrate material $(\varepsilon_{1}=6.2$), the calculated *C_S_* value was 4.59 nF, and then the calculated resonant frequency of the sensor was 108.15 MHz, which was very close to the measured value of 108.5 MHz, as shown in Figure 5b. It also showed that most of the calculation results had a good consistency with the measurement results, especially when the effective dielectric constant of the media filled in the microchannel was equal to or larger than the dielectric constant of LTCC substrate material. It suggested that the Maxwell–Garnett empirical equation was suitable for the calculation of the effective dielectric constant, of fluid-LTCC dielectric composites, in this work.

**Figure 6:**
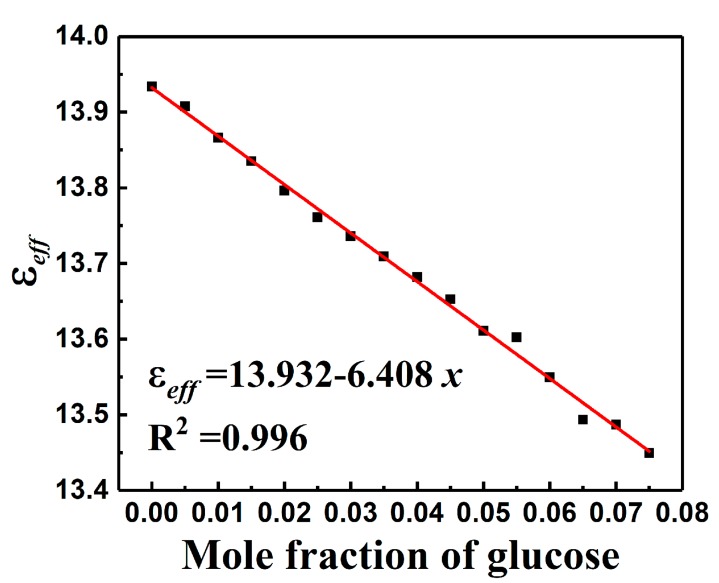
The relationship between the calculated effective permittivity and mole fraction of glucose.

The LTCC based wireless microfluidic sensors were further utilized to detect the concentration of glucose in water solution. Firstly, the dielectric properties of the glucose-water solution with mole fraction concentration (*x*) ranging from 0 to 0.075 were measured. The results are listed in Table 2. When glucose concentration was increased from 0 to 0.075, the permittivity gradually decreased from 78.11 to 62.43, while the dielectric loss gradually increased from 0.0757 to 0.465. The effective permittivity of the glucose-water solution calculated by Equations (6) and (7), as a function of the glucose mole fraction, is shown in Figure 6. The regression analysis indicated a good linear dependency ($R^{2}=0.996$) of the $\varepsilon_{eff}$ to the glucose mole fraction as $\varepsilon_{eff}=13.932-6.408x$. When glucose concentration increased from 0 to 0.075, the value of the calculated $\varepsilon_{eff}$ reduced by a small range. The variation of $\varepsilon_{eff}$ eventually led to the shift of $f_{r}$, making it feasible for the detection of glucose concentration. 

**Figure 7:**
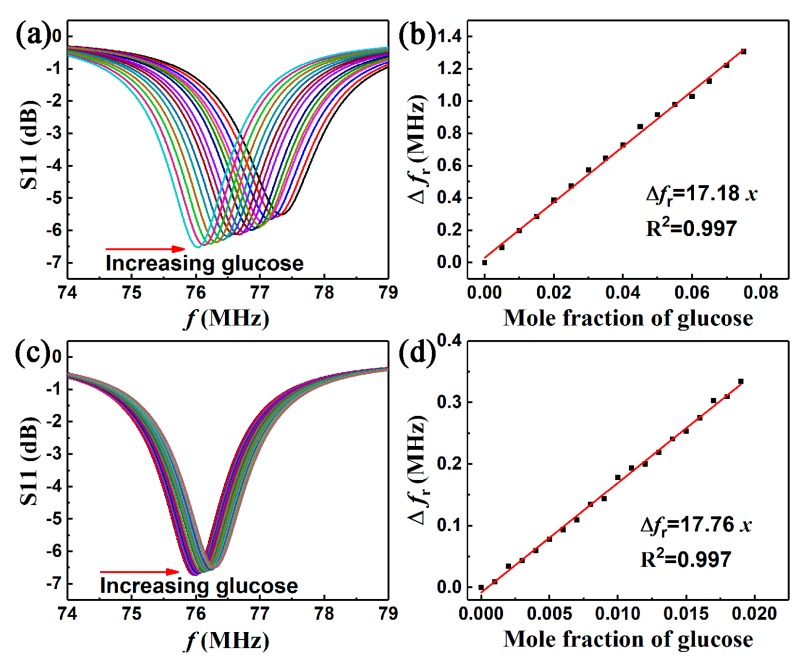
Measured frequency response of the LC wireless microfluidic sensor for the glucose-water solution. (**a**) Mole fraction concentration change equal to 0.005, and (**b**) the corresponding linear relationship with $\Delta f_{r}$, (**c**) glucose mole fraction concentration change equal to 0.001, and (**d**) the corresponding linear relationship with $\Delta f_{r}$.

Figure 7 shows the response of the wireless sensor to the glucose-water solution with various concentrations. $f_{r}$ increased from 76.028 to 77.335 MHz when the concentration (*x*) of glucose ranged from 0 to 0.075, as shown in Figure 7a. The measured resonant frequency shift was 1.307 MHz. Figure 7b shows a good linear relationship ($R^{2}=0.997$) between $\Delta f_{r}$ and the mole fraction concentration of glucose, $\Delta f_{r}=17.18x$. It proved the feasibility of detecting glucose in water solution through the shift of the resonant frequency. The amplitude of S11 gradually decreased with increases in glucose concentration. The same phenomenon has been reported previously [34,38], in which it was attributed to the increase in dielectric loss of the glucose-water solution. The glucose-water solutions, which had mole fractions ranging from 0 to 0.02, were tested to verify repeatability and detection resolution. The result in Figure 7c shows that $f_{r}$ increased with a similar slope to that shown in Figure 7a. A good linear dependency ($R^{2}=0.997$) of $\Delta f_{r}$ to the mole fraction of glucose was also obtained, as shown in Figure 7d. This indicated a good quantitative relationship between the shift of resonant frequency and the mole fraction of glucose-water solution. All in all, the sensor exhibited long-term reusability for wirelessly and accurately detecting glucose in water solutions through shifts in resonant frequency. The detection resolution was lower than 0.001. The sensor response $\Delta f_{r}/(f_{r}\cdot x)$ could be as high as 22%. 

Compared with the reported LTCC microfluidic biosensor based on electrochemical methods, absorption photometry, microwave resonators, and capacitive sensing with interdigitated capacitors (IDCs) [39,40,41,42,43], we provided a wireless detection method without using fluorescent labeling, complex chemical reactions, and wire connections. Moreover, the designed sensor was more sensitive to changes in the permittivity of liquids within a wide range of permittivity than most of the reported microwave electromagnetic sensors. Although only a limited number of non-polar and weak polar liquids were tested, the excellent reusability, non-invasiveness, fast responses, in addition to its chemical resistance, ease of constructing complex 3-D structures, and functional integration make this kind of LTCC based LC wireless microfluidic sensor very promising for wide range of chemical liquids analysis. Furthermore, it has great potential for establishing reliable wireless lab-on-a-chip for point-of-care, chemical, and environmental applications.

## 5. Conclusions

A novel LC wireless microfluidic sensor was demonstrated utilizing LTCC technology. The sensor was capable of sensitively detecting different liquids according to permittivity variation. The quantitative relationship between the resonant frequency of the developed sensor and the permittivity of the liquids was established. The sensor exhibits excellent reusability for accurately detecting water-glucose solutions with concentration as low as 0.1% mol. This kind of LTCC based, wireless microfluidic sensor would be a very promising platform for wireless lab-on-a-chip, bio-sensing, and real-time water and chemical analysis. 

## Figures and Tables

**Table 1:** Designed parameters of the sensor.

Symbol	Designed Value (mm)
a	9.6
d_0_	16.0
d_i_	10.4
s	0.40
w	0.40
t_1_	0.22
t_2_	0.86
t_3_	0.04
t_4_	0.39
d	1.60

**Table 2:** Dielectric properties of the glucose-water solution (20 °C, 500 MHz).

Mole Fraction of Glucose	$\varepsilon^{\prime }$	$\varepsilon^{\prime \prime }/\varepsilon^{\prime }$
0	78.11	0.0757
0.005	77.11	0.1207
0.010	75.56	0.1418
0.015	74.43	0.165
0.020	73.05	0.1821
0.025	71.85	0.2014
0.030	71.01	0.2306
0.035	70.13	0.2573
0.040	69.26	0.2852
0.045	68.34	0.313
0.050	67.05	0.3338
0.055	66.79	0.3816
0.060	65.23	0.4008
0.065	63.64	0.4284
0.070	63.46	0.4452
0.075	62.43	0.465
